# Cross-generational bacterial strain transfer to an infant after fecal microbiota transplantation to a pregnant patient: a case report

**DOI:** 10.1186/s40168-022-01394-w

**Published:** 2022-11-10

**Authors:** Shaodong Wei, Marie Louise Jespersen, Simon Mark Dahl Baunwall, Pernille Neve Myers, Emilie Milton Smith, Jens Frederik Dahlerup, Simon Rasmussen, Henrik Bjørn Nielsen, Tine Rask Licht, Martin Iain Bahl, Christian Lodberg Hvas

**Affiliations:** 1 National Food Institute, Technical University of Denmark, Kemitorvet 202, 2800 Kgs Lyngby, Denmark; 2 Clinical-Microbiomics A/S, Copenhagen, Denmark; 3 Novo Nordisk Foundation Center for Protein Research, Faculty of Health and Medical Sciences, University of Copenhagen, Copenhagen, Denmark; 4 Department of Hepatology and Gastroenterology, Aarhus University Hospital, Aarhus, Denmark; 5 Institute of Clinical Medicine, Aarhus University, Aarhus, Denmark

**Keywords:** *Clostridioides difficile* infection, Engraftment, Fecal microbiota transplantation, Gut microbiota, Infant, Neonatal seeding, Pregnancy, Strain transfer

## Abstract

**Background:**

Fecal microbiota transplantation (FMT) effectively prevents the recurrence of *Clostridioides difficile* infection (CDI). Long-term engraftment of donor-specific microbial consortia may occur in the recipient, but potential further transfer to other sites, including the vertical transmission of donor-specific strains to future generations, has not been investigated. Here, we report, for the first time, the cross-generational transmission of specific bacterial strains from an FMT donor to a pregnant patient with CDI and further to her child, born at term, 26 weeks after the FMT treatment.

**Methods:**

A pregnant woman (gestation week 12 + 5) with CDI was treated with FMT via colonoscopy. She gave vaginal birth at term to a healthy baby. Fecal samples were collected from the feces donor, the mother (before FMT, and 1, 8, 15, 22, 26, and 50 weeks after FMT), and the infant (meconium at birth and 3 and 6 months after birth). Fecal samples were profiled by deep metagenomic sequencing for strain-level analysis. The microbial transfer was monitored using single nucleotide variants in metagenomes and further compared to a collection of metagenomic samples from 651 healthy infants and 58 healthy adults.

**Results:**

The single FMT procedure led to an uneventful and sustained clinical resolution in the patient, who experienced no further CDI-related symptoms up to 50 weeks after treatment. The gut microbiota of the patient with CDI differed considerably from the healthy donor and was characterized as low in alpha diversity and enriched for several potential pathogens. The FMT successfully normalized the patient’s gut microbiota, likely by donor microbiota transfer and engraftment. Importantly, our analysis revealed that some specific strains were transferred from the donor to the patient and then further to the infant, thus demonstrating cross-generational microbial transfer.

**Conclusions:**

The evidence for cross-generational strain transfer following FMT provides novel insights into the dynamics and engraftment of bacterial strains from healthy donors. The data suggests FMT treatment of pregnant women as a potential strategy to introduce beneficial strains or even bacterial consortia to infants, i.e., neonatal seeding.

Video Abstract

**Supplementary Information:**

The online version contains supplementary material available at 10.1186/s40168-022-01394-w.

## Background

The commensal bacterial community residing in the human intestinal tract has attracted much attention in recent years and may represent a novel therapeutic target in various disease states [1]. Disturbances of this microbial ecosystem, e.g., by oral exposure to antibiotics, may disrupt the normal function of the microbiota potentially leading to acute expansion of opportunistic pathogenic bacteria [2]. A clinically important example of this is antibiotic-associated development of *Clostridioides difficile* infection (CDI), which accounts for approximately half a million infections per year in the USA alone [3] and thus represents a major healthcare challenge. Indeed, antibiotic treatment of infections carries an intrinsic risk of acquiring CDI, especially in vulnerable patient groups. This also explains the high recurrence rates observed following guideline-adherent treatment of patients with CDI, using vancomycin or fidaxomicin [4, 5]. In contrast, fecal microbiota transplantation (FMT) has proven exceptionally efficient to treat recurrent CDI with success rates ranging between 85 and 95% [6–10], underpinning the large potential of aiming at the microbiota as a therapeutic target, e.g., by re-establishing a rich and diverse ecosystem. The mode of action for FMT remains incompletely understood, but the transfer and possible engraftment of a microbiota from a healthy donor to a patient may prevent the recurrence of *C. difficile* via colonization resistance or competition for nutrients, as well as through the production of bacteriocins, secondary bile acids, short-chain fatty acids, or other metabolites [11, 12]. Additionally, donor-derived bacteriophages that are transferred to the recipient may also be involved [13]. Although consistent and defined bacterial consortia for CDI treatment are not yet available [14, 15], some beneficial species are likely mediators of a successful FMT [16], including members from families Ruminococcaceae, Lachnospiraceae, Bacteroidaceae, Rikenellaceae, and Porphyromonadaceae [17–20].

Previous studies have shown that long-term engraftment of bacterial communities (a transferable core gut microbiota) in the recipient may occur following FMT in patients with CDI [21, 22]. A recent study utilized metagenomic sequencing to track bacterial strains in 13 longitudinal clinical FMT interventions and reported that 71% of donor microbiota strains persisted in the recipients 5 years after the FMT and concurrently 80% of pre-FMT strains were seemingly eradicated from the recipient microbiota [22]. These observations highlight the potential of establishing a stable intestinal bacterial community in a patient with CDI, which to some extent results from transfer and engraftment in adult life. A consequence of this may be further transfer from the gut environment to other sites, including the vertical transmission of donor-specific strains to future generations during birth and early life intestinal microbiota establishment—as the mother’s gut is the major source of an infant’s intestinal microbiota [23]. Such transfer could potentially also function as a novel approach to facilitate neonatal seeding, by first modulating the gut microbiota of the pregnant mother by FMT and then allowing natural transfer to the new-born, e.g., of important infant associated *Bifidobacterium longum* strains [24].

Pregnancy is considered a contraindication for FMT in most guidelines [25], and studies of FMT during pregnancy are scarce [6]. Only a single clinical case report has been published, reporting the successful use of FMT during pregnancy for the treatment of CDI [26]. For this reason, vertical transmission of FMT donor-derived strains from the recipient patient to the newborn child has not previously been investigated. It is intrinsically challenging to unequivocally identify and track specific strains from the fecal donor as such strains may be very similar to conspecific strains already present in the patient before the FMT, which due to antibiotic treatment and/or CDI may be below the detection limit. A series of metagenomic tools have previously been developed to perform strain analysis either based on de novo assembling, reference database alignment, or statistical modeling of allele frequencies [27]. Conspecific strain resolution requires higher sequencing depth than species-level detection, which poses a challenge as there is generally little overlap in the species that are abundant in the adult and infant breastfed gut. We therefore developed and benchmarked a highly sensitive method based on allele frequencies that enable us to track engrafting strains in small sample populations.

In the present case report, we demonstrate the vertical transfer of donor strains to a recipient with later further transfer to the newborn infant. Based on the strain-level variation across individuals, we monitored the potential microbial transfer from the feces donor to the patient and further to the infant by identifying single nucleotide variants (SNVs) in the metagenomic samples.

## Materials and methods

### The patient and the medical history

**Fig. 1:**
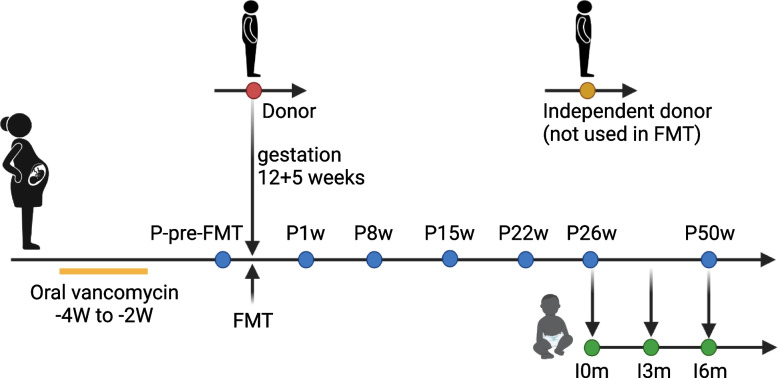
Timeline indicating vancomycin treatment, FMT, sampling time points for the mother and infant. Sampling time was relative to FMT (for the patient) or time of birth (for the infant). Colors indicate samples from donor (red), patient (blue, with samples taken before FMT (P-pre-FMT) and at 1, 8, 15, 22, 26, and 50 weeks after FMT), infant (green, with samples taken at birth (I0m) and at 3 and 6 months after birth), and an independent donor not used for the specific FMT (yellow). The patient vaginally delivered a healthy child at term, i.e., 26 weeks after the FMT

The patient was a Danish Caucasian woman, 29 years of age at the time of FMT treatment, and with no previous history of CDI. She became sick with diarrhea after having received oral V-Penicillin 2 mill IE three times/day (Vepicombin® Novum, Takeda Pharma A/S, Taastrup, Denmark) for 3 days and metronidazole 500 mg three times per day for periodontal disease. One month later, she became pregnant, and shortly after, she started to show gastro-intestinal symptoms, including frequent soft and liquid stools. She was prescribed metronidazole for 8 days, but she failed to recover. A positive PCR test of *C. difficile* toxin-producing genes *tcdA* and *tcdB* [28] confirmed the diagnosis of CDI. Second-line treatment with vancomycin 125 mg four times per day for 2 weeks conferred transient improvement, but clinical symptoms relapsed less than a week after cessation. Sixteen days after the vancomycin treatment ended, the patient was therefore administered a single colonoscopic FMT treatment at the Department of Hepatology and Gastroenterology at Aarhus University Hospital (AUH), at a gestation time of 12 + 5 weeks. The patient recovered, had an uneventful pregnancy course, and later gave term birth to a healthy infant (Fig. 1 and Fig. S1).

### Fecal microbiota transplantation

The anonymous feces donor was recruited from the Public Blood Center at AUH. The donated feces was thoroughly screened for pathogens as previously described [29] and was mixed with sterile saline, filtered to remove large particles, mixed with glycerol, and stored at − 80 °C in portions in cryobags following standard procedures [30]. The FMT was administered in the lower gastrointestinal tract with a flexible tube (colonoscope) inserted into the rectum and reaching the terminal ileum. The fecal suspension was infused into the terminal ileum, cecum, and ascending colon. A donor feces suspension originating from approximately 50 g of fresh feces was transplanted during the FMT. After the FMT, the patient was observed for 1 h and thereafter with scheduled outpatient follow-up.

### Fecal samples

Fecal samples were collected from the patient during active CDI before the FMT (P-pre-FMT) and at 1 week (P1w), 8 weeks (P8w), 15 weeks (P15w), 22 weeks (P22w), 26 weeks (P26w, at term), and 50 weeks (P50w, 6 months after giving birth) after the FMT (Fig. 1).

Fecal samples were also collected from the infant at birth (I0m, meconium), 3 months old (I3m), and 6 months old (I6m). The donor fecal material was also used for analysis. A fecal sample from a healthy donor, not used for the FMT of the patient, was included in the analysis and named “independent donor.” In total, twelve fecal samples were collected and investigated by shotgun metagenomic sequencing. Furthermore, metagenomic data from 651 healthy 1-year-old Danish infants [31] and 58 healthy Danish adults [32] were included for comparison purposes.

### DNA extraction and sequencing

DNA was extracted from 0.18–0.22 g of feces content of each sample with the DNeasy® PowerLyzer® PowerSoil® isolation kit (Qiagen) according to the manufacturer’s recommendations. The concentration of the extracted DNA was determined by fluorometric Quantification (Qubit 2.0 Fluorometer, Thermo Fisher Scientific) and stored at − 20 °C until use. Library preparation was preformed using the Nextera XT DNA Library Preparation Kit (Illumina, Int., San Diego, CA, USA) according to the manufacturer’s recommendation, by the DTU in-house facility (DTU Multi-Assay Core (DMAC), Technical University of Denmark). Size confirmation of the target was performed on a Bioanalyzer DNA 1000 chip (Agilent Technology, CA), and the DNA concentration was determined with Qubit 2.0 Fluorometer. DNA libraries were mixed in equimolar ratios. Sequencing was performed as a 150-bp paired-end run on HiSeq 3000/4000 (Illumina Int., San Diego, CA USA) at Novogene Europe’s facility following the manufacturer’s recommendations.

### Screening for *Clostridioides difficile* toxin genes

Analysis of fecal samples for the presence of *C. difficile* toxin genes was performed with SYBR-green-based quantitative PCR (qPCR). We used primers targeting the *C. difficile* toxin genes *tcdA* (F: AAT TTA GCT GCA GCA TCT GAC ATA G, R: TTC CCA ACG GTC TAG TCC AAT AG) and *tcdB* (F: GGA GAA TGG AAG GTG GTT CA, R: CTG GTG TCC ATC CTG TTT CC). The PCR reactions each contained 5.5 μl LightCycler® 480 II SYBR Green I Master (Roche Diagnostics A/S, Hvidovre, Denmark), 0.2 μM of each primer, and 2 μl template DNA in a total reaction volume of 11 μl. Reaction conditions were as follows: initial 95 °C for 5 min, followed by 45 cycles of 95 °C for 10 s, 60 °C for 15 s, and 72 °C for 45 s. Finally, a melting curve was generated by gradually increasing the temperature (95 °C for 5 s, 68 °C for 1 min and increasing the temperature to 98 °C with a rate of 0.11 °C/s with continuous fluorescence detection).

### Gene catalog and metagenomic species definitions

As a reference gene catalog, we used the Clinical Microbiomics Human Gut HG04 gene catalog (14,355,839 genes), which was created based on 12,170 non-public deep-sequenced human gut specimens (including 481 from infants), 9428 publicly available metagenomes compiled from 43 countries [33], and 3567 publicly available genome assemblies from isolated microbial strains [34–36]. For taxonomic abundance profiling, we used the Clinical Microbiomics human-gut MGS catalog (HGMGS version HG4.D.1) with a set of 2095 metagenomic species (MGS), each represented by a set of genes with highly coherent abundance profiles and base compositions across the 12,170 metagenomes. The metagenomic species concept has been described previously [37].

To taxonomically annotate an MGS, we blasted its genes against the NCBI RefSeq genome database (2020-01-27) and used rank-specific annotation criteria. Specifically, we assigned a taxon to an MGS if at least *M* % of its genes were mapped to the taxon and no more than *D* % of its genes were mapped to a different taxon. We only considered blast hits with an alignment length ≥ 100 bp, ≥ 50% query coverage, and % identity ≥ *PID*. Here, we define *PID* = (95, 95, 85, 75, 65, 55, 50, 45); *M* = (75, 75, 60, 50, 40, 30, 25, 20); and *D* = (10, 10, 10, 20, 20, 20, 20, 15) for subspecies, species, genus, family, order, class, phylum, and superkingdom, respectively. Finally, we processed each MGS with CheckM [38] and updated our annotation with the CheckM result if this resulted in a lower taxonomic rank.

### Sequencing data preprocessing

Raw FASTQ files were filtered to remove host contamination by discarding read pairs in which either read mapped to the human reference genome GRCh38 with Bowtie2 (v.2.3.4.1) [39]. Reads were then trimmed to remove adapters and bases with a Phred score below 20 using AdapterRemoval (v.2.2.4) [40]. Read pairs in which both reads passed filtering with a length of at least 100 bp were retained; these were classified as high-quality non-host (HQNH) reads. We obtained an average size of 9.0 ± 4.4 (mean ± standard deviation) Gbases for HQNH reads and achieved coverages ranging from 79.8 to 99.0% based on the Nonpareil 3 [41] (Fig. S2).

### Mapping reads to the gene catalog

HQNH reads were mapped to the gene catalog using BWA mem (v.0.7.16a) [42]. An individual read was considered mapped to a gene if the mapping quality (MAPQ) was ≥ 20 and the read aligned with ≥ 95% identity over ≥ 100 bp. However, if > 10 bases of the read did not align to the gene or extend beyond the gene, the read was considered unmapped. Reads meeting the alignment length and identity criteria but not the MAPQ threshold were considered multi-mapped.

Each read pair was counted as either (1) mapped to a specific gene, if one or both individual reads mapped to a gene; (2) multi-mapped, if neither read was mapped, and at least one was multi-mapped; or (3) unmapped, if neither individual read mapped. If the two reads each mapped to a different gene, the gene mapped by read 1 was counted but not the gene mapped by read 2. A gene count table was created with the number of mapped read pairs for each gene.

### MGS relative abundance calculation

For each MGS, a signature gene set was defined as the 100 genes optimized for accurate abundance and phylogenetic profiling of the MGS using a proprietary method. In short, signature genes are selected to be omnipresent among conspecific strains and distinct from any other gut organism, including uncharacterized species. Moreover, the genes show an even abundance-dependent mapping across 12,170 samples thereby allowing the detection of low abundant MGS. An MGS count table was created by counting the number of reads mapped to the MGS signature genes per sample. An MGS was considered detected if reads from a sample mapped to at least three of its signature genes; measurements that did not satisfy this criterion were set to zero. Based on internal benchmarks using a set of samples down sampled to 10 million and subsequently to 250,000 reads, this threshold of 3 genes results in 99.6% specificity when comparing MGS detection in the shallow sample to the signal in the deep sample. The relative abundance of each MGS was calculated by normalizing the MGS count table according to effective gene length and then normalized sample-wise to sum to 100%.

Down-sampled (rarefied) MGS abundance profiles were calculated by random sampling, without replacement, of a fixed number of signature gene counts per sample, and then following the procedure described above. In this study, 71,081 signature gene counts were sampled.

### Phylogeny

SNVs were identified from all the alignments between sequencing reads and MGS-specific signature genes. BCFtools multiallelic-caller (v.1.11) was used to extract information about alleles for each position of the signature genes for the subset of alignments with a MAPQ ≥ 30 [43, 44]. Bases with a base calling quality < 13 were filtered. All alternative alleles were kept while indels were filtered. The phylogenetic tree for an MGS was built based on the SNV data. For each MGS, the major strain DNA sequences across the 100 MGS signature genes were identified in the samples with at least 250 reads mapping to the signature genes and where at least 10 (10%) of the signature genes were detected. For each position along a signature gene, reads calling the reference allele and any other alternative allele (SNV). A minimum depth of at least 2 reads was required to call a position. If multiple alleles were observed for a position, 98% of the reads had to call the same allele. If the allele frequency was below 98%, the position was said to be mixed and no allele was called. If more than 20% of the covered positions in a gene were mixed in a sample, the entire gene was unknown (corresponding to “N”) for that sample. The sample-specific inferred DNA sequences across the 100 signature genes were combined, and sequences were combined across samples to produce one multiple sequence alignments. The position which was not called in any sample (corresponding to “N”) were trimmed. Phylogenetic trees were inferred using IQtree2 (v.2.1.2) [45, 46]. ModelFinder was used to select a substitution model for each of the genes [47].

All trees were rooted using an outgroup of two MGS. The outgroup was selected as the two MGS that contained the highest number of genes that were homologous to the signature genes in the target MGS. Homologous genes (marker genes) shared between MGSs were identified by annotating all MGS genes at the protein level to PFAM and TIGRFAM protein families based on the homology search using INTERPROSCAN (v.5.50-84.0) [48]. Genes that were annotated to more than one PFAM or TIGRFAM family were excluded. Protein families detected more than once in an MGS were also filtered. Once the marker genes had been identified, these were aligned at the protein level using MAFFT (v.7.453) [49, 50]. The protein alignment was translated back to the nucleotide.

### Strain level profiling

For tracking of strains, we used an approach implemented at Clinical Microbiomics. The investigations were based on the identified signature gene SNVs from each sample for the MGSs that were detected in at least one infant sample. To track donor strain transfer to the patient and later to the infant, two different methods were implemented. Initially, we tried to distinguish between strains of the same species found in the patient prior to the FMT (P-pre-FMT) and in the donor, by identifying the polymorphic positions between the two samples (P-pre-FMT and donor). To reduce the effect of sequencing errors, these positions were filtered to have at least two reads coverage and an allelic frequency of at least 98%, which is in line with a previous study [51]. If 50 or more polymorphic positions could be found between the two strains, the depths of each of the polymorphic positions were summed for all the other samples from the patient and infant. Furthermore, a total of 50 or more reads mapping to these positions were required for looking at the proportion of the different strains within a sample. We will refer to this part of the engraftment profiling as the discriminative positions method (Fig. S3A).

Alternatively, when the pre-FMT strain could not be identified, either because it was absent or had insufficient data, we estimated the distance between the post-FMT strain and the donor strain and related it to phylogenetic distances estimated from gut microbiome samples from two Danish cohorts to detect strain transfers. The expected variation within each species was identified by creating phylogenetic trees for MGSs based on 709 metagenomic samples from healthy, Danish infants (*n* = 651) and adults (*n* = 58). The 1% percentile of all phylogenetic distances within the tree was identified and used as the maximum percentage of allowed dissimilarity between the same strains to account for the fact that different species evolve at different rates [52, 53]. Phylogenetic distances correspond to the number of mutation events per base and are therefore not necessarily the same as the observed number of dissimilarities but likely close. The observed dissimilarities between the donor and other samples, corresponding to “1—the average nucleotide identity (ANI),” were calculated based on all positions for an MGS with at least 2 reads coverage and 98% allelic frequency within a sample. Samples with an overlap of at least 1000 positions with the donor sample were included in the analysis. The donor strain was expected to be transferred to a sample if the fraction of dissimilar positions were smaller than the 1% percentile of all phylogenetic distances. This part of the engraftment profiling is referred to as the percentage of identical alleles method (Fig. S3B).

To validate the results found with the two different strain profiling methods, we created an artificial dataset with samples containing two strains (DSM 22959 [GCF_008000975.1] and JCM 30893 [GCF_009731575.1]) of *Akkermansia muciniphila* isolated from human feces. The whole genomes of these two strains were downloaded from NCBI, and the average nucleotide identity (ANI) between the two strains was assessed to be 98.69% using fastANI (v.1.1) [54]. Reads of 150 bp were simulated from the genomes with wgsim from samtools (v.1.9) [55], setting mutation rate and indel fraction to zero but using the default base error rate. FASTQ files were simulated with wgsim at 20 times the coverage of one of the strains. bbmap (v.38.9) [56] reformat was used to downsize the FASTQ files to different coverages (20×, 10×, 4×, 2×, and 1×) and percentages (0%, 1%, 2%, 5%, 10%, 30%, 50%, 70%, 90%, 95%, 98%, 99%, or 100%) of the strains. The FASTQ files from the two different strains were combined to contain a total of 100% within the same coverage. We applied both strain profiling methods to this dataset to evaluate their performance.

### Analysis of short-chain fatty acids

The short-chain fatty acids (SCFAs) in fecal samples were profiled with a gas chromatography-mass spectrometry (GC-MS) method specially targeted to SCFAs using a high polarity column. Briefly, 250–500 mg of feces was diluted with three times the volume (weight [g]/volume [μL]) of PBS (i.e., 750–1500 μL) and vortexed to make a slurry. Then, samples were centrifuged three times at 16,000 *g* for 30 min. Thereafter, the supernatant was filtered through Costar SpinX centrifuge filters 0.2 μm at the recommended top speed of 15,000 *g* for 2 min until clear. The obtained fecal water was stored at − 20 °C before analysis at MS-Omics (Vedbæk, Denmark). For analysis, samples were acidified using hydrochloride acid, and deuterium-labeled internal standards were added. All samples were analyzed in a randomized order. Analysis was performed using a high polarity column (Zebron™ ZB-FFAP, GC Cap. Column 30 m × 0.25 mm × 0.25 μm) installed in a GC (7890B, Agilent) coupled with a quadropole detector (5977B, Agilent). The system was controlled by ChemStation (Agilent). Raw data was converted to netCDF format using Chemstation (Agilent), before the data was imported and processed in Matlab R2014b (Mathworks, Inc.) using the PARADISe software described by Johnsen et al. [57].

## Results

### Clinical outcomes

Following the application of the FMT, all CDI-related symptoms (including frequent soft and liquid stools) alleviated within 24 h, and no adverse effects related to the FMT were observed. The *C. difficile* toxin genes *tcdA* and *tcdB* were detected in the patient’s feces prior to FMT (P-pre-FMT), but not in feces samples 1 week after the FMT nor at any subsequent sampling points (Fig. S4). No recurrence of CDI-related symptoms was recorded during the follow-up. The infant was fully developed and vaginally delivered at term and without complications. The infant was exclusively breastfed until 4 months of age, when porridge and infant formula were gradually introduced alongside continued breastfeeding. The infant had no clinical signs of CDI during the study period, although both *tcdA* and *tcdB* genes were detected by qPCR at the age of 6 months (Fig. S4). Consistent with this observation, *C. difficile* was also detected by metagenomic sequencing in samples P-pre-FMT (relative abundance 7.9 × 10^−6^) and I6m (6.7 × 10^−5^).

### Gut microbiota composition differences between the donor, patient, and infant

**Fig. 2:**
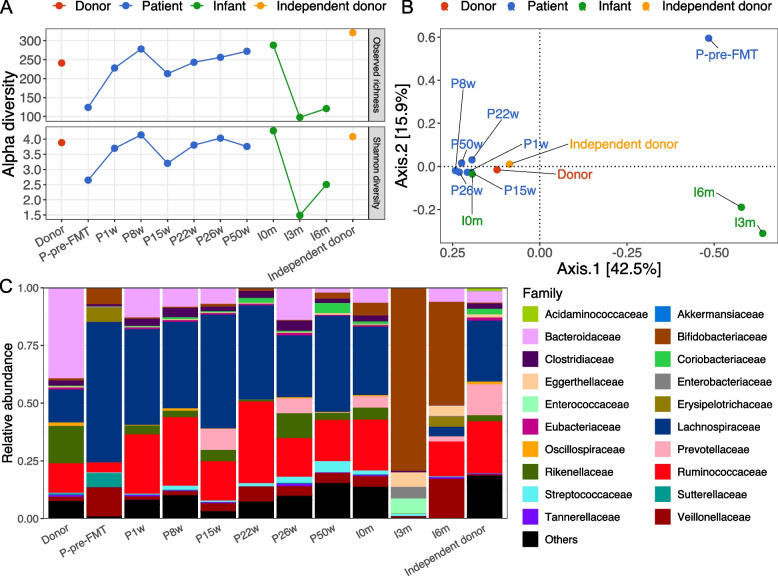
Gut microbiota compositional differences between the donor, patient, and infant. **A** Alpha diversity (observed richness and Shannon diversity) for all subjects. **B** Beta diversity of all subjects based on the Bray-Curtis distance and visualized with principal coordinates analysis (PCoA). **C** The taxonomic composition for the top 20 families. Less abundant families were merged as “Others.” Fecal samples were collected from the donor and an independent (not-used) donor as well as from the patient during active CDI before the fecal microbiota transplantation (P-pre-FMT) and at 1 week (P1w), 8 weeks (P8w), 15 weeks (P15w), 22 weeks (P22w), 26 weeks (P26w, at term), and 50 weeks (P50w, 6 months after giving birth) after the FMT. Fecal samples were collected from the infant at birth (I0m, meconium), 3 months old (I3m), and 6 months old (I6m)

The patient pre-FMT displayed a lower species/MGS richness than the donor and all later patient sampling time points (Fig. 2A). The infant at 3 and 6 months of age displayed even lower microbial richness, but the richness at birth was similar to that of the mother (Fig. 2A). A similar pattern was observed for Shannon diversity (Fig. 2A). To assess the changes in overall gut microbial community composition, we estimated the beta diversity of gut microbiota at the species/MGS level based on Bray-Curtis distances visualized by principal coordinate analysis (PCoA) (Fig. 2B). The patient pre-FMT was well separated from all the post-FMT samples and the donor, which was also the case for the infant sampled at both 3 and 6 months of age. Notably, the patient post-FMT samples were clustered together with those from the donor, the independent donor, and the infant at birth (meconium) (Fig. 2B).

Next, we depicted the gut microbiota composition at the family level (Fig. 2C). Compared with the donor, the patient pre-FMT had marked lower relative abundances of Bacteroidaceae, Ruminococcaceae, Rikenellaceae, and higher relative abundances of Lachnospiraceae, Bifidobacteriaceae, Erysipelotrichaceae, Sutterellaceae, and Veillonellaceae. However, after FMT, the depleted family pre-FMT were largely restored. The restoration of gut microbiota composition in the patient post-FMT could also be evidenced by the highly consistent microbial differences in genus-level abundances between the patient pre-FMT and the donor, in comparison with the differences before and after FMT (average of all post-FMT patient samples) (Fig. S5). The independent donor showed a different microbial composition compared to that of the actual donor, especially for families Bacteroidaceae and Rikenellaceae, which likely contributed to the distance in the ordination (Fig. S6).

Accompanying the microbiota shift, SCFAs also showed differential concentrations across samples (Fig. S7). The fecal concentration of most SCFAs was low in the patient before FMT compared with the donor and after the FMT treatment, i.e., butyrate, propionate, and valerate.

We further assessed the top 50 species having the highest variance in the relative abundances in the donor and patient before and after FMT (Fig. S8). Many of these species were abundant in patient pre-FMT and depleted in the donor and patient post-FMT, or vice versa. The patient pre-FMT carried higher relative abundances of putatively pathogenetic species, such as *Clostridium innocuum* (associated with extra-intestinal clostridial infection) [58], *Clostridium symbiosum* (associated with colorectal adenocarcinoma) [59], *Clostridium clostridioforme* (involved in bacteremia) [60], *Veillonella parvula* (producing lipopolysaccharide, which is the main virulence factor that leads to meningitis) [61], *Flavonifractor plautii* (associated with colorectal cancer) [62], and *Ruminococcus gnavus* (associated with Crohn’s disease) [63].

### Transfer of microbes at the species level between the donor and patient

**Fig. 3:**
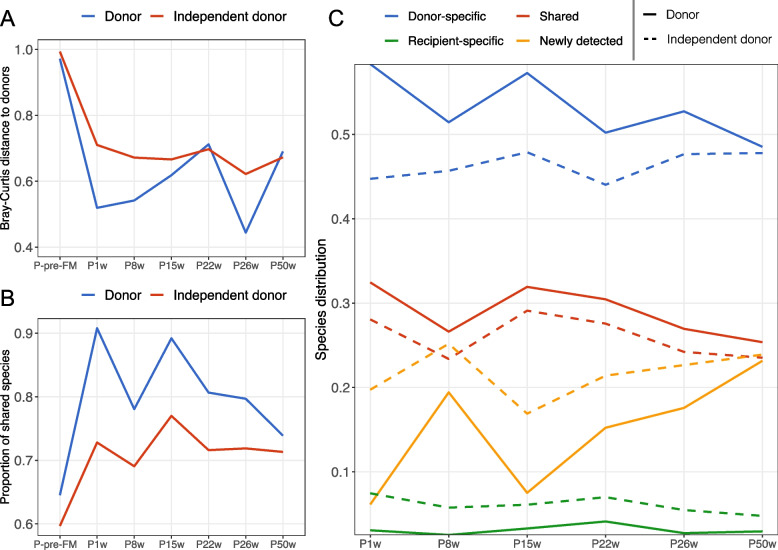
Transfer of donor microbes to the patient, estimated at the species level. **A** The Bray-Curtis distance between the patient and the donor or the independent (not-used) donor over time. **B** The proportion of species common to the patient and the donor or independent donor over time. **C** Contributions to the patient post-FMT gut microbiota. Possible sources were divided into four categories, namely donor-specific (species from the donor and not shared with the patient pre-FMT), shared (species common to donor and the patient pre-FMT), recipient-specific (species from the patient pre-FMT and not shared with the donor), and newly detected (species not included in the above categories, possibly from the environment or below the detection limit). Line types indicate the measurement was performed with the donor (solid) or the independent donor (dashed). Fecal samples were collected from the donor and an independent (not-used) donor as well as from the patient during active CDI before the fecal microbiota transplantation (P-pre-FMT) and at 1 week (P1w), 8 weeks (P8w), 15 weeks (P15w), 22 weeks (P22w), 26 weeks (P26w, at term), and 50 weeks (P50w, 6 months after giving birth) after the FMT

We further assessed the gut microbiota dissimilarity between the patient and the actual donor or the independent donor over time at the species/MGS level (Fig. 3A). The calculated Bray-Curtis distance between donor and patient was very large pre-FMT but was reduced dramatically 1 week after FMT and thereafter underwent a gradual increasing trend until week 50, except for an unexplained reduction at week 26. Compared with the donor used for FMT, the independent donor generally had larger Bray-Curtis dissimilarities to the patient (Fig. 3A). Next, we estimated the proportion of shared species between the patient and the donor over time (Fig. 3B). Before the FMT, the patient had 64.5% of species common to the donor, while after FMT, the proportion increased to 90.8% at week 1 and remained higher albeit with a decreasing trend until 50 weeks after the FMT. The independent donor shared much fewer species with the patient (Fig. 3B).

To investigate the potential contribution sources of the bacteria identified in the patient after FMT, we stratified the patient post-FMT gut microbiota into four categories, namely shared, donor-specific, recipient-specific, and newly detected (Fig. 3C, Fig. S9). We found that donor-specific species were the highest in percentage and accounted for 58.3% at week 1 and roughly remained stable during the study period. The following contributions were the shared, newly detected, and recipient-specific species (Fig. 3C). In comparison, when the independent donor was used in place of the actual donor in these analyses, the proportions of donor-specific and shared species were lower at all sampling time points. Also, we followed the transferred donor-specific species after FMT and found that over 80% of these could be successfully transferred and maintained until 50 weeks (Fig. S10), much higher than the independent donor. Due to the transfer of donor-specific species, the patient post-FMT was very different from the baseline (P-pre-FMT) in the gut microbiota composition based on the Bray-Curtis dissimilarity (> 0.970) (Fig. S11), which is consistent with the results in Fig. 2B.

Although the donor-specific species represented the largest proportion in number, their abundances were low (Fig. S12) and were largely from the families Bacteroidaceae, Lachnospiraceae, Clostridiaceae, and Ruminococcaceae. A full list of these transferred donor-specific species is shown in Fig. S13. Because the species with the highest abundances were present in both donor and pre-FMT samples, we were unable to detect transfer at the species level for bacteria; therefore, a deeper level investigation of microbial transfer was necessary.

### Transfer of microbes at the strain level between the donor, patient, and infant

**Fig. 4:**
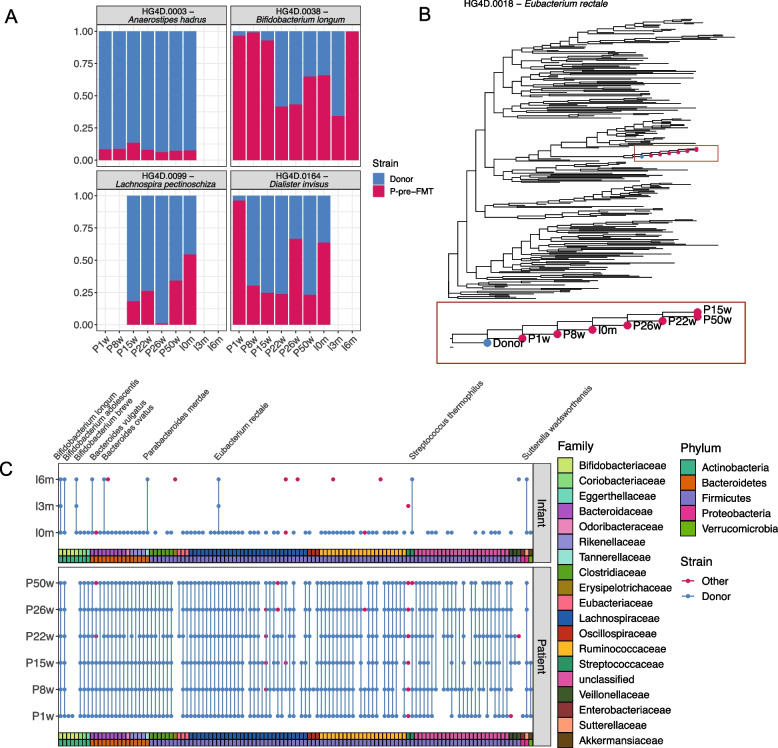
Transmission of donor strains to the patient and maternal transmission to the infant. **A** The proportion of donor and pre-FMT patient strains from four different species detected in the infant at birth and 3 and 6 months after birth, based on discriminative positions. **B** The phylogenetic tree for HG4D.0018—*Eubacterium rectale* containing 246 strains identified from publicly available adult and infant samples from healthy Danes. Donor and patient samples are highlighted in blue and red, respectively. The phylogenetic tree highlighted in the box is the zoom of the bigger tree. **C** Detection of donor strain in patient and infant samples across 120 different species based on the percentage of identical alleles between strains identified in the donor and in the patient or infant samples. Fecal samples were collected from the FMT donor and an independent (not-used) feces donor as well as from the patient during active CDI before the FMT (P-pre-FMT) and at 1 week (P1w), 8 weeks (P8w), 15 weeks (P15w), 22 weeks (P22w), 26 weeks (P26w, at term), and 50 weeks (P50w, 6 months after giving birth) after the FMT. Fecal samples were collected from the infant at birth (I0m, meconium), 3 months old (I3m), and 6 months old (I6m)

Due to subspecies heterogeneity in the human microbiome [53], the transfer of microbes was further investigated at the strain level based on the tracking of SNVs in the metagenomic data. Using these SNVs we were able to identify donor strains in two different ways. The first method (discriminative positions) estimated the relative abundance of the donor as well as the patient pre-FMT strain for those species that had sufficient coverage in both the P-pre-FMT and donor strain to identify discriminative SNVs between them (Fig. 4A). When a species was detected in the donor sample but not in the P-pre-FMT sample or when it was too low abundant in the P-pre-FMT sample to identify discriminatory SNVs, the donor strains were tracked using the SNV-similarity between the post-FMT strain and donor strain in comparison to its similarity to strains identified from other, unrelated individuals (percentage of identical alleles, example of the phylogeny base of this method in Fig. 4B). We found that both methods performed well, when evaluated on a mock dataset (Fig. S14).

When looking at the percentage of identical alleles, we could investigate the strain transfers for 120 different species, which was close to half of all the species (241) detected in the donor sample. Of these 120 species, 116 of them were also detected at the species level in one or more of the patient samples, and 99% of them (115/116 species) had strain transfer from the donor to the patient (Fig. 4C, Fig. S15). In addition, we observed alternative strains in one or more patient samples for 14 of the species (Fig. S16). In the infant samples, we were able to detect 87 different species and found donor strain engraftment in 92% of these (80 species). However, of these 80 species, 87.5% (70 species) were only found in the meconium sample and much fewer donor strains were detected in the infant’s gut at later time points (3.8% [3/80] in the 3-month sample and 12.5% [10/80] in the 6-month infant sample) (Fig. S17). Interestingly, we found that when strains of the same species co-existed in the mother after the treatment, both strains were transmitted to the infant as well (Fig. 4A).

Collectively, the strain transfers identified based on discriminative alleles (Fig. 4A) were few but consistent and to some extent included in the strain transfers identified based on the percentage of identical alleles (Fig. 4C). For example, the strain transfer in the species *Bifidobacterium longum* was confirmed by both methods, thus underscoring an actual transfer event. Out of the 115 species where the donor strains showed engraftment in the mother, 82 were also detected at the species level in at least one infant sample. For 79 of these 82 species engrafting the mother, the donor strain was also detected in the infant, indicating that most of the donor strains (96.3%) were transferred from mother to infant, although this is mostly based on the detection of donor strains in the meconium sample. Interestingly, we were able to detect a *Bifidobacterium breve* donor strain across all infant samples, even though we never detected it in the mother. *B. breve* is associated with the breastfed microbiome [24], and it is therefore expected that its abundance would be higher in the infant than in the mother. Co-existence of both the donor and maternal pre-FMT derived *B. longum* subsp. *longum* strains was observed in the infant. At 3 months of age, the donor strains were the dominant strain in the infant, while the maternal pre-FMT strain had become the vastly dominant strain at 6 months of age (Fig. 4A). Additional details for strain transfer findings are available in the supplementary material Section S1.

## Discussion

The FMT treatment of the pregnant CDI patient reported here was successful and deemed necessary to ensure the health of both the patient and the unborn child. In addition to curing the patient, the procedure uniquely allowed us to investigate the potential cross-generational transfer of donor-derived microbiota from donor to patient and further to the infant. We demonstrate, at the strain level and for the first time, the vertical transfer of donor fecal bacteria administered during routine FMT from a recipient mother to her later-born infant. This was achieved by analyzing SNV patterns from the metagenomic samples. Our results suggest the frequent presence of donor strains in both the patient samples (99% of donor strains) and infant meconium (92% of donor strains). Furthermore, the finding of a donor strain (*B. breve*) in the infant that was not detected in the mother suggests that some strains transferred from the donor remained below the detection level in the mother but were still passed on at birth (Fig. 4C). Interestingly, the dynamics of the *B. longum* strains originating from either patient (P-pre-FMT) or donor (Fig. 4A) indicated a dominance of the donor-derived strain in the infant at 3 months, during exclusive breastfeeding, and later a shift to almost complete dominance of the mother derived strain at 6 months. The observed “strain sweep” may be linked to the introduction of complementary foods. The lower level of donor strain detection in the infant samples at 3 and 6 months after birth, compared to meconium, could be explained by a high ecological selection pressure in the infant gut, e.g., due to human milk oligosaccharides (HMOs) [64], and only strains with suitable fitness will be present at detectable abundances. Also, it cannot be ruled out that physical contamination of meconium samples could have occurred during the sampling process but perinatal transfer from the mother during vaginal birth seems most likely [65]. Nevertheless, we were still able to detect donor strains in 12.5% of species in the infant samples at 6 months of age, indicating frequent strain transfer and engraftment. The relatively high level of donor-derived strains in the infant at 6 months demonstrates the frequent cross-generational transfer of newly introduced bacterial strains in the mother, considering the potential influences of maternal microbial dysbiosis on the newborn [66], it indicates FMT as a potential strategy for neonatal seeding for instance FMT or probiotic treatment before delivery could normalize maternal microbiota before birth and avoid “unhealthy” strain/community transfer to the infant. Such an approach would however need further validation in larger studies and should also include in-depth safety assessments of the donor-derived bacterial consortia to avoid long-term consequences of engrafting and spreading potentially problematic strains in the gut community of the infant. The gut microbiome has been suggested to further mature until the age of 2.5 years [67]; thus, more long-term studies of donor strain engraftment in the child would be interesting to investigate in future studies.

Regarding the CDI, we observed substantial differences in the gut microbiota composition between the patient and the healthy donor. The gut microbiota in the patient pre-FMT was characterized by low alpha diversity (Fig. 2A) and depletion of members in families Bacteroidaceae, Ruminococcaceae, and Rikenellaceae (Fig. 2C, Fig. S5), which is consistent with previous reports [17–20]. Following FMT, the gut microbiota of the patient was normalized to a healthier status, which was evidenced by the increased alpha diversity and reduced dissimilarity to the donor (Fig. 2A, B). The patient pre-FMT was observed to harbor a reservoir of potential pathogens (Fig. S8); however, these pathogens were largely reduced by the FMT and thereafter represented similar abundances as in the donor. Together, this suggests that FMT successfully normalized the gut microbiota structure and potentially inhibited the growth of pathogens [12]. After FMT, the depleted families such as Bacteroidaceae, Rikenellaceae, and Ruminococcaceae were largely restored (Fig. 2C, Fig. S5), likely by direct transfer and engraftment (Fig. 3, Fig. S12). These families together with Lachnospiraceae have been suggested to be positively associated with FMT response [18, 68] and included strains, such as *Bacteroides*, *Dorea*, *Roseburia*, *Alistipes*, and *Parabacteroides* are beneficial for a successful FMT, potentially due to their ability to produce short-chain fatty acids [16, 18, 69]. Consistently, we observed that FMT led to an overall increase in SCFAs (Fig. S7). Especially, the restoration of valerate by FMT potentially inhibited the growth of *C. difficile* and may have contributed to the clinical resolutions [70].

## Conclusions

We here present evidence for strain transfer from an FMT donor to a pregnant patient and further to her newborn baby. The findings substantiate the possibility that introduced exogenous strains in the gut environment during FMT treatment may propagate across generations potentially facilitating a novel approach for modulating the intestinal microbiota.

## Supplementary Information

**Additional file 1: Fig. S1.** Medical history for the patient from onset of CDI symptoms to FMT treatment and delivery of an infant. **Fig. S2.** The estimated genome coverage based on the Nonpareil. **Fig. S3.** Illustrations of the strain detection methods. **Fig. S4.** Detection of the C. difficile toxin producing genes with qPCR. **Fig. S5.** Tree plots to show the differences in the taxa relative abundances at different taxonomic levels. **Fig. S6.** Beta diversity of donors and patient post-FMT. **Fig. S7.** Short-chain fatty acids detected in the fecal samples. **Fig. S8.** Heatmap to show the top 50 species having the largest variance across donor and patient samples. **Fig. S9.** A diagram to show the division of the gut microbiota in the patient post-FMT. **Fig. S10.** Proportion of transferred donor-specific species. **Fig. S11.** Bray-Curtis distance to show the dissimilarity between the patient post-FMT and the patient pre-FMT. **Fig. S12.** The relative abundances of species in the patient post-FMT when divided into different potential sources. **Fig. S13.** Heatmap to show the donor-specific species in the patient gut after FMT. **Fig. S14.** Benchmarking of discriminative positions method and percentage of identical alleles method. **Fig. S15.** Detected donor strains in patient and infant samples across 120 different species based on the percentage of identical alleles between strains identified in the donor and in the patient or infant samples. **Fig. S16.** Detection of donor strain in the 14 species with one or more samples with another strain detected. **Fig. S17.** Changes in the number of species detected to have strain transfer from the donor.

**Additional file 2: Section S1.** Additional details for strain transfer findings.

## Data Availability

The datasets supporting the conclusions of this article are available in the National Center for Biotechnology Information (NCBI) with the Sequence Read Archive (SRA) bioproject number PRJNA812583 (https://www.ncbi.nlm.nih.gov/bioproject/PRJNA812583).
