# Supplementary material for: Cross-generational bacterial strain transfer to an infant after fecal microbiota transplantation to a pregnant patient: a case report
Source: Microbiome. 2022 Nov 10;10:193. doi: 10.1186/s40168-022-01394-w (PMC9647999; doi:10.1186/s40168-022-01394-w)
Supplement: Supplementary file 2 — Additional file 1: Fig. S1. Medical history for the patient from onset of CDI symptoms to FMT treatment and delivery of an infant. Fig. S2. The estimated genome coverage based on the Nonpareil. Fig. S3. Illustrations of the strain detection methods. Fig. S4. Detection of the C. difficile toxin producing genes with qPCR. Fig. S5. Tree plots to show the differences in the taxa relative abundances at different taxonomic levels. Fig. S6. Beta diversity of donors and patient post-FMT. Fig. S7. Short-chain fatty acids detected in the fecal samples. Fig. S8. Heatmap to show the top 50 species having the largest variance across donor and patient samples. Fig. S9. A diagram to show the division of the gut microbiota in the patient post-FMT. Fig. S10. Proportion of transferred donor-specific species. Fig. S11. Bray-Curtis distance to show the dissimilarity between the patient post-FMT and the patient pre-FMT. Fig. S12. The relative abundances of species in the patient post-FMT when divided into different potential sources. Fig. S13. Heatmap to show the donor-specific species in the patient gut after FMT. Fig. S14. Benchmarking of discriminative positions method and percentage of identical alleles method. Fig. S15. Detected donor strains in patient and infant samples across 120 different species based on the percentage of identical alleles between strains identified in the donor and in the patient or infant samples. Fig. S16. Detection of donor strain in the 14 species with one or more samples with another strain detected. Fig. S17. Changes in the number of species detected to have strain transfer from the donor. [file 40168_2022_1394_MOESM1_ESM.docx]

**Supplementary material**

**Cross-generational bacterial strain transfer to an infant after fecal microbiota transplantation to a pregnant patient: a case report**

Shaodong Wei, Marie Louise Jespersen, Simon Mark Dahl Baunwall, Pernille Neve Myers, Emilie Milton Smith, Jens Frederik Dahlerup, Simon Rasmussen, Henrik Bjørn Nielsen, Tine Rask Licht, Martin Iain Bahl, Christian Lodberg Hvas

**Supplementary figures**

**
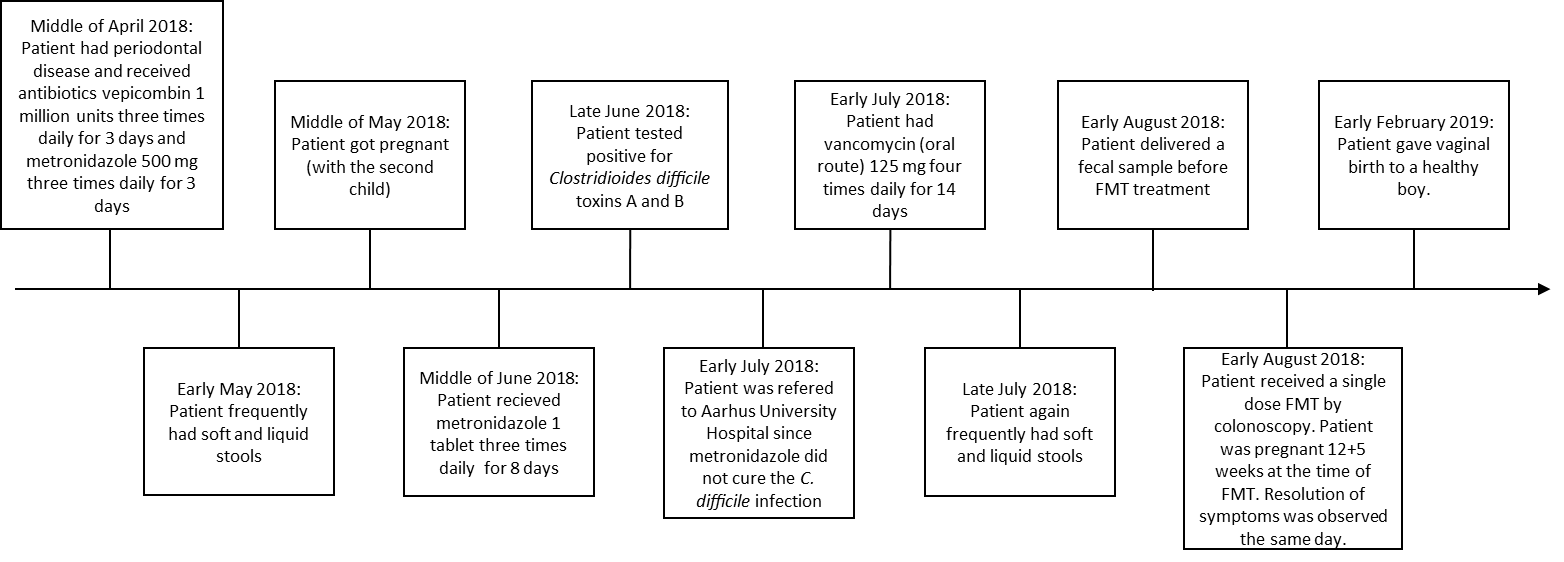
**

**Fig. S1. Medical history for the patient from onset of CDI symptoms to FMT treatment and delivery of an infant.**

**Fig. S2. The estimated genome coverage based on the Nonpareil.** The X axis is the sequencing effort (bp) needed to reach a corresponding coverage in Y axis. Each line refers to a sample. The dashed red line is the 95% coverage.

**Fig. S3. Illustrations of the strain detection methods.** (A) The discriminative positions method. The donor and pre-FMT discriminative alleles are quantified in the patient samples after FMT. Each position has at least 2 reads coverage and an allelic frequency of at least 98%. At least 50 discriminative positions are required to define a strain. The relative abundance of a strain originated from the donor or pre-FMT is calculated based on the number of donor or pre-FMT specific reads. (B) The percentage of identical alleles method. The expected strain variation is calculated based on phylogenetic trees of MGSs from 709 metagenomic samples from healthy Danish infants (n = 651) and adults (n = 58). Phylogenetic distances correspond to number of mutation events per base and are therefore not necessarily the same as the observed number of dissimilarities but likely close. The observed dissimilarities between the donor and other samples, corresponding to “1 - the average nucleotide identity (ANI)”, was calculated based on all positions for an MGS with at least 2 reads coverage and 98% allelic frequency within a sample. Samples with an overlap of at least 1,000 positions with the donor sample were included in the analysis. The donor strain was expected to be transferred to a sample if the fraction of dissimilar positions were smaller than the 1% percentile of all phylogenetic distances.


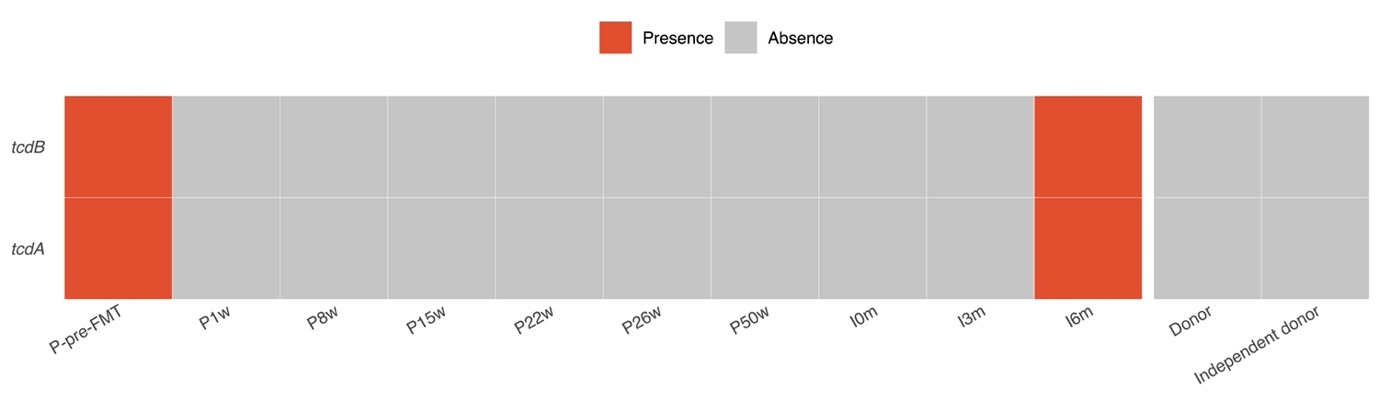


**Fig. S4. Detection of the *C. difficile* toxin producing genes with qPCR.** The toxin genes *tcdA* and *tcdB* were detected with SYBR green based qPCR. The patient before FMT and the six months old infant showed positive results.

**Fig. S5. Tree plots to show the differences in the taxa relative abundances at different taxonomic levels.** (A) The comparison of differences between the patient pre-FMT and the donor. (B) The comparison of differences between the patient pre-FMT and the patient post-FMT. For (A) and (B), the smallest nodes represent the genus level and the node in the center represents the kingdom bacteria. Node sizes are proportional to the mean relative abundance at the given phylogenetic level. Colors refer to comparison of relative abundances, where the blue color indicates the taxa are more abundant in the patient pre-FMT, red color indicates taxa are more abundant in the donor or the patient post-FMT. Node labels and colors above family level are not shown.

**Fig. S6. Beta diversity of donors and patient post-FMT.** The beta diversity was assessed with the Bray-Curtis distance and visualized with principal coordinates analysis (PCoA).

**Fig. S7. Short-chain fatty acids detected in the fecal samples.** The Y axis is the abundance of short-chain fatty acids (SCFAs) in the unit of micromole per gram. The Y value zero indicates a SCFA was below the detection limit.

**Fig. S8. Heatmap to show the top 50 species having the largest variance across donor and patient samples.** Colors refer to the relative abundance (log10 transformed for visualization) of species. The annotation colors on the left side refer to the families and phyla where a species is from.

**
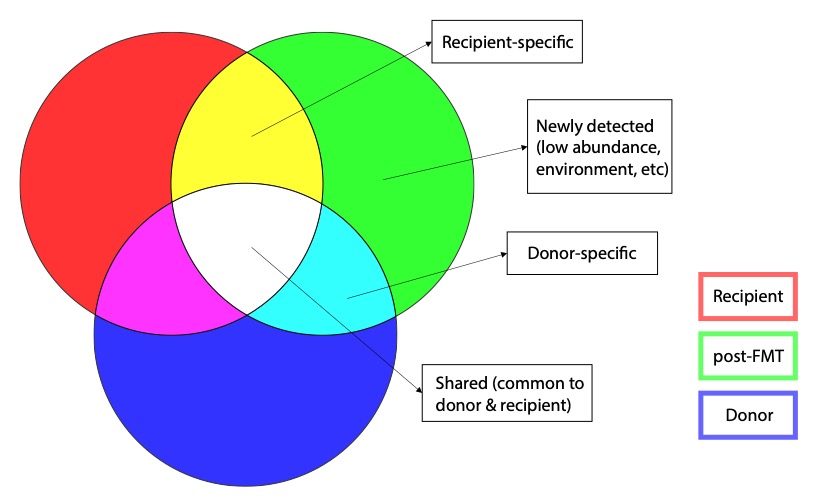
**

**Fig. S9. A diagram to show the division of the gut microbiota in the patient post-FMT.** The gut microbiota in the patient post-FMT is categorized into four categories, namely shared (species common to the donor and the patient pre-FMT), donor-specific (species from the donor and not shared with the patient pre-FMT), recipient-specific (species from the patient pre-FMT and not shared with the donor), and newly detected (species not included in the above three categories, possibly from the environment or below detection limit).

**Fig. S10. Proportion of transferred donor-specific species.** We define here the donor-specific species as the species that are from the donor but not shared with the patient pre-FMT. The Y axis is the proportion of donor-specific species that are successfully transferred in the patient gut (formulated as the area proportion in **Fig. S9**: cyan/[cyan + blue]). The species from the real donor (used for FMT) represented around 80% of the post-FMT patient gut microbiota. In contrast, species from the unrelated donor (used as a comparison) accounted for less than 40%.

**Fig. S11. Bray-Curtis distance to show the dissimilarity between the patient post-FMT and the patient pre-FMT.** For the sake of interpretation, distance close to 1 indicates that the patient was very different from the baseline in terms of the gut microbiota composition.

**Fig. S12.** **The relative abundances of species in the patient post-FMT when divided into different potential sources.** Species are colored at the family level. Only the top 20 families are shown and the remaining was merged as “Others”. The gut microbiota in the patient post-FMT is categorized into four categories (more details in **Fig. S9**), namely shared (species common to the donor and the patient pre-FMT), donor-specific (species from the donor and not shared with the patient pre-FMT), recipient-specific (species from the patient pre-FMT and not shared with the donor), and newly detected (species not included in the above three categories, possibly from the environment or below detection limit).

**Fig. S13. Heatmap to show the donor-specific species in the patient gut after FMT.** Donor-specific species was defined as species from the donor and not shared with the patient pre-FMT (more details in **Fig. S9**). Colors refer to the relative abundance of species. The annotation colors on the left side refer to the families and phyla where a species is from.

**Fig. S14. Benchmarking of the strain methods: discriminative positions (A) and overlapping positions (B).** The methods were benched marked using artificial sequence reads generated from the two *Akkermansia muciniphila* (HG4D.0110) strains: DSM 22959 and JCM 30893, refered to here as “donor” and “other”. Random reads from these artificial sequence reads dataset where then combined to create artificial mixtures of the two strains at 0/100, 1/99, 2/98, 5/95, 10/90, 30/70, 50/50, 70/30, 90/10, 95/5, 98/2, 99/1, and 100/0 of DSM 22959 and JCM 30893, respectively. For each ratio/mixture of the two genomes multiple mixtures were generated so the total coverage was 1x, 2x, 4x, 10x, and 20x. (A) Scatter plots showing the expected vs. the observed mixture of the two strains. Discriminative positions between the strains were identified at the coverages 1x, 2x, 4x, 10x, and 20x. The number of discriminative positions between DSM 22959 and JCM 30893 using the data with 1x and 2x coverage in the donor sample was lower than the minimum threshold of 50 (3 and 11 positions, respectively). For the remaining coverages in the donor sample, 4x, 10x, and 20x the accuracy of the method was compared across samples with coverages of 1x, 2x, 4x, 10x, and 20x (indicated in the grey bar on top of scatter plots). Accuracy was calculated as the R-squared value. (B) Plots showing whether the strain in the mixture was identified as being either the donor (here donor is the reference strain) or other at the different ratios of mixtures of the two strains (rows) and with different total coverages in the sample (columns). This was done for five different coverages (1x, 2x, 4x, 10x, and 20x) in the reference donor sample as well, indicated on top of each plot.


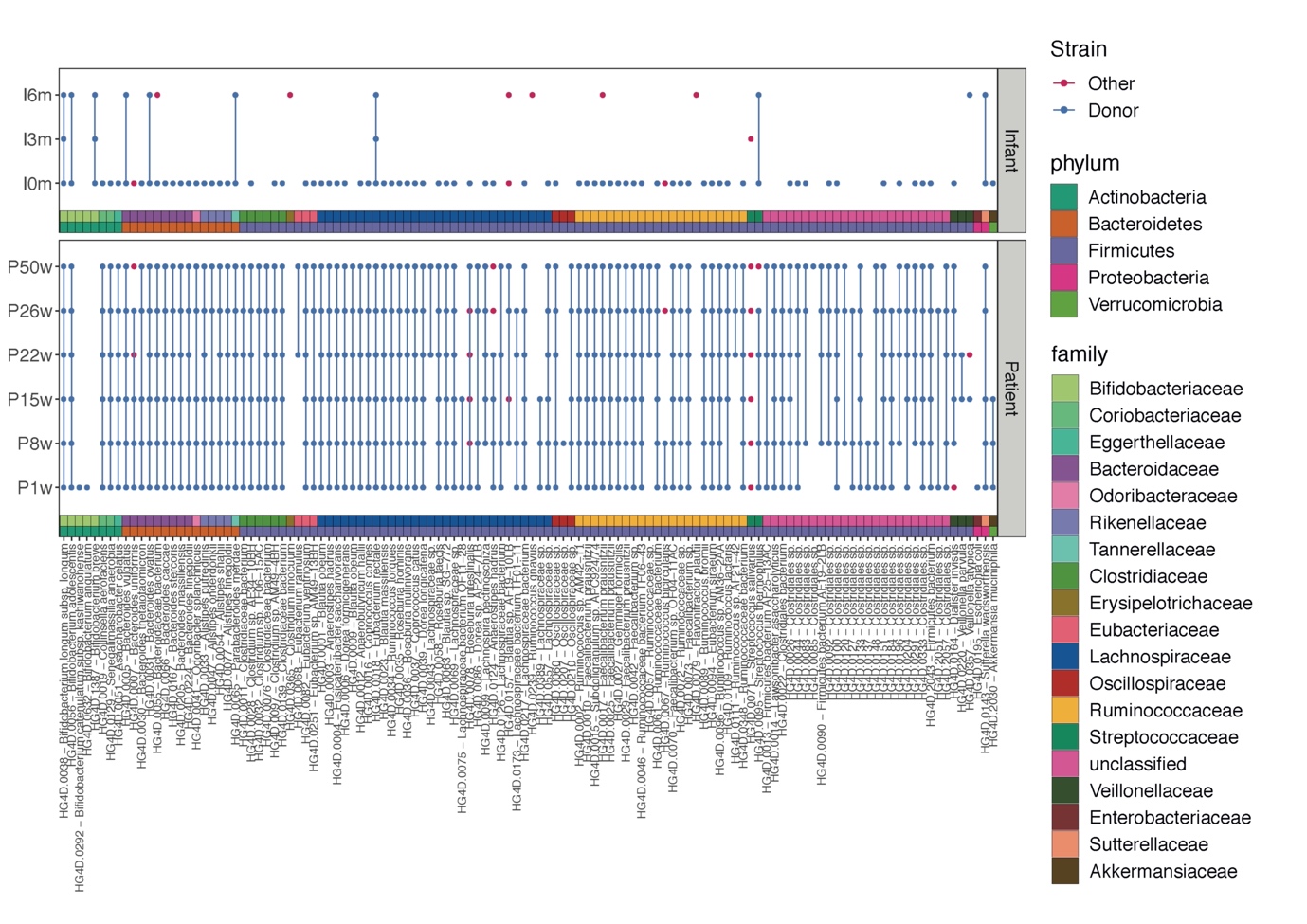


**Fig. S15. A full list of transferred strains across 120 different species based on the percentage of overlapping positions between strains identified in the donor and in the patient or infant samples.**

**Fig. S16. Detection of donor strain in the 14 species with one or more samples with another strain detected.** Extract of the plot from Fig. 4C, including only 14 species for which another strain than the donor strain was detected in one or more samples. Colors illustrate whether the donor strain was identified in a sample.

**Fig. S17. Changes in the number of species detected to have strain transfer from the donor.** The X axis is the samples from the patient (not including P50w) and the infant over time. The Y axis is the number of species with strain transfer. In total 120 species were detected to have strain transfer. This figure is a different presentation of **Fig. 4C**. These 120 species in the patient or infant were classified into “Donor” (species with strains coming from the donor), “Other” (species with strains coming from unknown sources), “Not available” (species could not be detected, either because it was absent or had insufficient data).
