# Supplementary material for: Cross-generational bacterial strain transfer to an infant after fecal microbiota transplantation to a pregnant patient: a case report
Source: Microbiome. 2022 Nov 10;10:193. doi: 10.1186/s40168-022-01394-w (PMC9647999; doi:10.1186/s40168-022-01394-w)
Supplement: Supplementary file 3 — Additional file 2: Section S1. Additional details for strain transfer findings. [file 40168_2022_1394_MOESM2_ESM.docx]

**Section S1. Strain transfer details**

**Transfer of microbes at the strain level between the donor, patient, and infant**

By using two different strain profiling methods, in total, we could investigate strain transfers for 120 different species, which was close to half of all the species (241) detected in the donor sample. Of these 120 species, strain transfer from the donor to the patient was detected for 115 species (**Fig. 4C, Fig. S15**). Only one of the 120 species (*Streptococcus salivarius*) did not engraft in any of the samples from mother or infant, despite other strains of the species being detected across multiple time points. An additional four species (*Bifidobacterium breve*, *Clostridium innocuum*, *Ruminococcus gnavus*, *Flavonifractor plautii*) were only detected at sufficient depth to access transmission of donor strains in one or more infant samples, but not in any of the patient samples.

Overall, we detected alternative strains in one or more samples for 14 species (**Fig. S16**). For three of these (*F. plautii*, *R. gnavus*, and *C. innocuum*), the species was only detected in the infant sample taken at six months and never in the mother. This indicates that the donor strains did not engraft in the mother and as a result therefore could not be transferred to the infant. For three other species (*Bacteroides uniformis*, *Blautia sp. AF19-10LB*, and *Ruminococcus bicirculans*) where we did detect strain engraftment in the mother, we were also unable to detect strain transmission to the infant. For *R. bicirculans*, *Anaerostipes hadrus,* and *Streptococcus thermophilus*, the donor strain detected in the mother were either lost or were no longer the dominant strain by the time she gave birth or by the time the infant was six months old. Likewise, the two donor strains, *Bacteroidaceae bacterium* and *Faecalibacterium prausnitzii*, found in the infant at birth, were no longer detectable in the infant sample at six months.

For *B. uniformis* and *B. sp. AF19-10LB*, we detected alternative strains in maternal samples where we both in a previous and later sample detected the donor strain, indicating that strain mixing could result in our inability to detect donor strains, when they are not sufficiently abundant compared to alternative strains. Similar shifts in strain dominance were also observed for *Roseburia intestinalis* and *Dialister invisus*. For *D. invisus* the co-existence of donor and mother/recipient (P-pre-FMT) originated strains (strain mixing) was confirmed by the discriminative positions method. Additionally, strain mixing was also found for the three other species (*A. hadrus*, *Bifidobacterium longum,* and *Lachnospira pectinoschiza*) detected with this method (**Fig. 4A**). For *A. hadrus*, *L. pectinoschiza*, and *D. invisus*, strain transfer in the infant samples could only be detected in the sample at birth (meconium). However, for *B. longum*, strain mixing was still detected when the infant was three and six months old. Interestingly, despite the *B. longum* species being dominated by the strain originating from the donor in the infant at three months of age, the six months infant samples revealed that the maternal pre-FMT strain had then become vastly dominant, even though the mother harbored >25% of the donor specific strain at this time-point (**Fig. 4A**).

Among the species that contained enough polymorphic positions to discriminate between donor and pre-FMT strains, only four were sufficiently abundant in at least one infant sample to track the abundance of donor and maternal pre-FMT strains. The lack of overlapping species was probably a result of both low richness in the maternal pre-FMT sample (124 species in P-pre-FMT *vs.* 213-278 species in the post-FMT samples, **Fig. 2A**) and general very large differences in the species that colonize the infant and adult gut [1], with *B. longum* being a prominent species in the infant intestine.

Reference

1. Bäckhed F, Roswall J, Peng Y, Feng Q, Jia H, Kovatcheva-Datchary P, et al. Dynamics and Stabilization of the Human Gut Microbiome during the First Year of Life. Cell Host Microbe. 2015;17:690–703.
